# Infant Stimulation Induced a Rapid Increase in Maternal Salivary Oxytocin

**DOI:** 10.3390/brainsci12091246

**Published:** 2022-09-15

**Authors:** Kana Minami, Teruko Yuhi, Haruhiro Higashida, Shigeru Yokoyama, Takahiro Tsuji, Chiharu Tsuji

**Affiliations:** 1 Research Center for Child Mental Development, Kanazawa University, Kanazawa 920-8640, Japan; 2 Division of Socio-Cognitive-Neuroscience, United Graduate School of Child Development, Osaka University, Kanazawa University, Hamamatsu University School of Medicine, Chiba University and University of Fukui, Kanazawa 920-8640, Japan; 3 Department of Health Development Nursing, Institute of Medical, Pharmaceutical and Health Sciences, Kanazawa University, Kanazawa 920-0942, Japan; 4 Department of Ophthalmology, Faculty of Medical Sciences, University of Fukui, Fukui 910-1193, Japan

**Keywords:** oxytocin, saliva, serum, perinatal period, mother-infant interaction, breastfeeding, labor, pregnancy

## Abstract

Oxytocin (OT) is a neuropeptide involved in human social behaviors and reproduction. Non-invasive OT levels in saliva have recently roused interest as it does not require a specialized medical setting. Here, we observed one woman’s basal serum and saliva OT from pregnancy to 1 year postpartum to track OT concentration changes over this period. We examined the changes in salivary OT levels over time in response to maternal physiological and behavioral responses. The fluctuation of saliva OT levels is well correlated with serum OT during pregnancy and breastfeeding. However, while salivary OT increased rapidly during direct interaction (social interaction tests) with the infant and/or when the mother was watching her own infant’s video (video tests), no increase was observed in serum. We used social interaction and video tests on a group of mothers (nine mothers for social interaction and six for the video test) to clarify these single-subject results. In both tests, the mothers had increased OT in their saliva but not serum. Our study may suggest that salivary samples reflect not only the physical but also the emotional state and that saliva samples may be useful for monitoring women’s OT levels during pre- and postpartum periods. Further studies with larger sample numbers are necessary to confirm the rapid changes in salivary OT levels in response to maternal physiological and behavioral responses.

## 1. Introduction

Oxytocin (OT) is a neuropeptide implicated in social and reproductive behavior across many mammalian species including humans [1]. Mainly produced in the hypothalamus, OT has an important role both peripherally and centrally. In the peripheral, OT has long been known for its association with uterine contractions and lactation [1]. Centrally, it contributes to a wide range of social behaviors and social relationships, besides modulating stress- and anxiety-related behaviors [1,2]. Furthermore, high OT releases during the peripartum period to promote the mother-offspring bond and regulate maternal behavior such as care, offspring recognition, maternal aggression, and reduced anxiety and fear responses in rodents and sheep [3,4]. The amount and quality of parenting behaviors have been shown to correlate with OT signaling pathways in rodents, rhesus macaques, and humans [5], and the OT signaling pathways of offspring are highly modulated by their experience in youth in rhesus macaques and prairie voles [6,7]. In prairie voles, disrupted maternal care is reported as highly influential in the offspring’s social behaviors [8].

These OT roles in maternal behaviors and mother-child bonding are implicated in humans as well [9]. Mothers’ postpartum social behavior has been associated with OT in maternal plasma and saliva [10,11]. The interaction with the parent induces the infant’s OT release and the association between the parent’s and infant’s OT levels was greater under conditions of higher affect synchrony [12]. Plasma OT was associated with bonding-related cognition and increased activation of motivated limbic brain regions in response to infants’ stimulation [13,14]. By contrast, genomic modification in OT signaling-related genes correlates with the reduced quality of maternal behaviors [5,15,16]. For example, risk alleles of the *OXTR* and *CD38* genes, which are implicated in social dysfunctions, were associated with reduced amounts of parental touch toward their infants and parent-infant gaze synchrony [15]. Furthermore, correlating with animal study findings, the lack of emotional and physical contact from parents influences children’s OT system [17]. Maltreated children or children raised by a mother suffering from mental illness, i.e., postpartum depression, are reported to have dysregulated OT systems [18,19,20]. Accumulated evidence has shown that negative social relationships in youth have a lifelong influence on children and are likely to induce mental illness or inability in social adaptation [21,22,23,24]. Therefore, a deeper understanding is needed of the underlying physiology of normal and impaired mother-child interactions related to their OT systems and expanded association studies of OT signaling and maternal behaviors and/or bonding in humans.

Human OT levels have been measured in samples of plasma, serum, urine, or cerebrospinal fluid (CSF). Each collection method has advantages and disadvantages. CSF reflects central OT levels but requires a lumbar puncture, which is highly invasive and is only performed by physicians. Urine collection is less invasive and does not require professionals, but the temporal resolution is low, as it is in CSF collection. In comparison, blood collection requires a medical professional but is less invasive than CSF collection and has a much better temporal resolution than the other two sample types. Therefore, collecting plasma or serum has been the most commonly used method for detecting OT levels, especially in studies of women during perinatal periods [25]. However, blood draws may induce a stress response to needles, and a habituation period is necessary before sampling. Recently, saliva sampling has become a more popular way to measure OT levels [10,26,27,28,29,30]. The advantages of saliva sampling include its lack of need for a medical setting and its time resolution, which is better than a blood draw [31,32]. Thus, saliva sampling may overcome experimental settings’ limitations and reveal the dynamic changes in OT concentration in response to physiological stimuli or experimental settings that have not been detected in previous studies. 

The use of peripheral OT concentrations as an indicator of central concentrations is highly beneficial considering the invasive procedures required to collect CSF in humans. However, presently, an understanding of whether and under what conditions peripheral OT concentrations are used as an indicator of central OT is still lacking. In animals, it is possible to simultaneously perform blood sampling and brain microdialysis to compare peripheral and central OT release. Studies in rodents have shown that the release of OT into the brain and bloodstream can occur either cooperatively or independently with stimulation [33]. In breastfeeding, brain and blood OT concentration increased simultaneously [34]. With osmotic stimulation, peripheral OT concentrations increase before OT release in the brain [35]. Social defeat stress induces the release of brain OT but not in the periphery [36]. Alternatively, in humans, it has been argued that central and peripheral OT releases are functionally independent [37]. However, recent studies have demonstrated a correlation between central and peripheral OT concentration in patients who have neurological and neurosurgical diseases [38]. Patient samples revealed no correlation between plasma and CSF but did show a strong correlation between saliva and CSF. Carson et al. collected blood and CSF samples from patients undergoing clinically indicated lumber procedures or CSF-related procedures and found a positive correlation between them [39]. Further, both plasma and CSF OT concentrations negatively predicted anxiety scores. As it has been shown in rodent studies, the central and peripheral release of OT can occur either cooperatively or independently with stimulation in humans as well [40]. However, it is not easy to conduct an association study of CSF and peripheral OT concentrations in humans due to the highly invasive nature of CSF collection procedures. Thus, it is important to carefully examine which peripheral fluid, salivary or blood, is an efficient alternative to identify CSF oxytocin concentrations depending on the behavioral or emotional context.

In this study, we examined changes in OT levels in the saliva and serum of a woman in pre- and post-pregnancy. A woman’s hormonal balance alters drastically when she becomes pregnant, and dynamic changes occur in the mind and body while nurturing a child. However, not many studies collect saliva and plasma or serum simultaneously or investigate how OT concentrations fluctuate physiologically in women’s specific life periods [41]. Therefore, we conducted a trajectory study following one woman’s salivary and serum OT levels during pregnancy and labor. We also assessed OT concentration changes while breastfeeding, during direct mother-infant interaction (social interaction test), and while watching a video of their infant (video test). In addition, salivary and serum OT concentrations were evaluated in several mothers during social interaction and video tests. We hypothesized that salivary OT concentrations may fluctuate in a similar manner to serum during pregnancy, parturition, and lactation; however, they are uncoupled in the infant’s stimulation.

## 2. Materials and Methods

### 2.1. The Participant

The subject of the trajectory study was a Japanese woman in her 40′s, gravida 4 para 3, carrying a singleton pregnancy at the start of this study. She had a husband and a nuclear family. Her final education was a Ph.D., and she is a full-time employee. She has not been prescribed psychiatric medication or hormonal treatment and is in good health as assessed by the doctor and the nurse throughout this study period. The time required for delivery was approximately 7 h. The infant was delivered after 40 weeks, 3 days gestation, with a birth weight of 3393 g. At the end of the third trimester of delivery, 500 mL of Veen D mixed with 1 A unit of Antonin O5 was administered over approximately 2 h. Both mother and baby passed from conception to postpartum period with no abnormalities. On the third postpartum day, the blood draw was Hb 11.4/dL. The mother stayed in the same room with her child from the second postpartum day and fed the baby mainly by formula eight to nine times a day every 3 h. She was able to feed the infant solely with breast milk from the fifth postpartum day.

Ten Japanese mothers who were 6–7 months postpartum, had no problems with their progress from pregnancy to postpartum, and no history of mental illness were recruited for the social interaction test. Six of these mothers participated in the video and control (reading) test. For analysis, samples of mothers who scored within the interquartile range (IQR) of ± 1.50 in the EPDS test were used (3, median, 2.9 ± 1.97, average ± S.D.). The sizes of the dataset in the social interaction test and video/control test were nine and six, respectively. The mean age of the mothers in the social interaction test and video/control test were 32.3 ± 4.8 and 32.0 ± 5.3 (average ± S.D.) years old, respectively. All the children were born full-term (37–40 weeks) and weighed 2690–3620 g at birth. The experiment was conducted outside of the menstrual period for all participants. The demographic and obstetric characteristics of the participants are shown in Table 1.

This research was approved by Kanazawa University Medical Ethics Review Committee in 2019 (approval number 3096-2). All procedures were conducted in accordance with the ethical standards of the institutional research committee and with the 1964 Helsinki Declaration. The participants signed informed consent.

### 2.2. Blood and Saliva Collection Procedure

**Figure 1:**
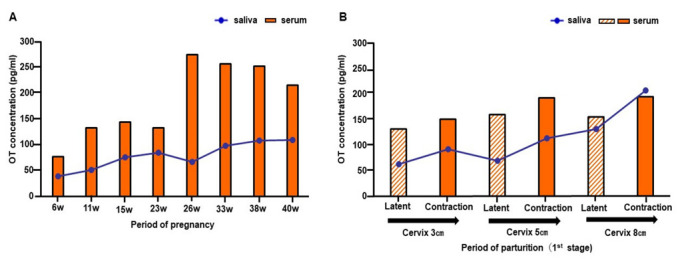
Changes in salivary and serum oxytocin (OT) from early pregnancy to the labor of one woman. (**A**) Salivary and serum OT concentrations from gestation weeks 6 to 40. (**B**) Salivary and serum OT concentrations in the first stage of labor.

**Figure 2:**
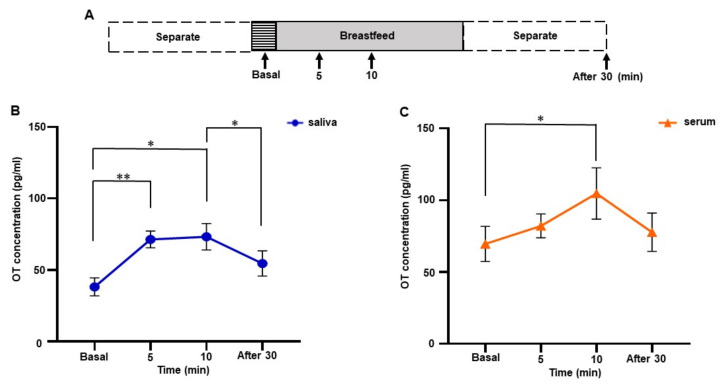
Changes in salivary and serum oxytocin (OT) concentrations during breastfeeding in one woman. (**A**) The time course of experiments. Samples were collected before (basal), 5, and 10 min after initiation of breastfeeding, and 30 min after feeding ended. Values were averaged from samples taken on postpartum days 1, 3, 5, 7, 30, and 90. (**B**) Salivary OT concentration. (**C**) Serum OT concentration. The experiment was repeated six times with one mother (n = 6). The values are the mean ± SEM. OT: oxytocin. One-way repeated measures ANOVA (d.f. = 3; * *p* < 0.05 ** *p* < 0.01).

**Figure 3:**
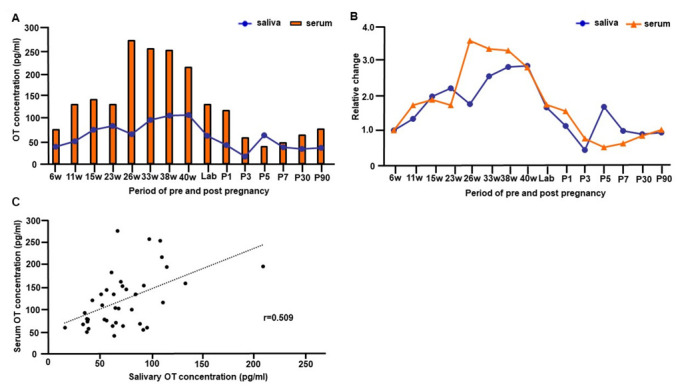
The trajectory of salivary and serum oxytocin (OT) concentrations from early pregnancy to 90 days postpartum of one woman. (**A**) The concentration of salivary and serum OT levels from 6 weeks gestation to 90 days postpartum. The basal OT concentration from the measurement of breastfeeding experiments was used for values of postnatal days. The value in the intermittent period of first stage labor was used for plotting the time point of labor (value at cervical opening 3). (**B**) The trajectory of relative changes of salivary and serum OT levels from 6 weeks gestation to 90 days postpartum. (**C**) Correlation between salivary and serum OT concentrations from early pregnancy to postpartum day 90. Note that OT concentrations from every time point of all postnatal breastfeeding experiments were plotted.

**Figure 4:**
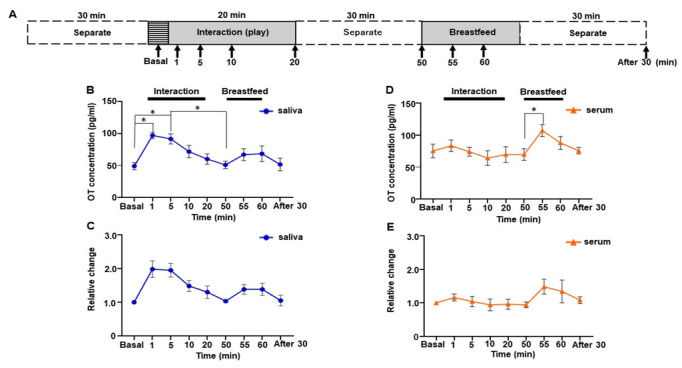
Changes in saliva and serum oxytocin (OT) concentrations during the direct mother-infant interaction test of one woman. (**A**) Schematic procedure of the interaction test. (**B**) Saliva OT concentration. (**C**) Relative changes in salivary OT. (**D**) Serum OT concentration. (**E**) Relative change in serum OT. The experiment was repeated six times with one mother (n = 6). Values are the means of six repeated experiments and are the means ± SEM; one-way repeated measures ANOVA (d.f. = 8; * *p* < 0.05).

**Figure 5:**
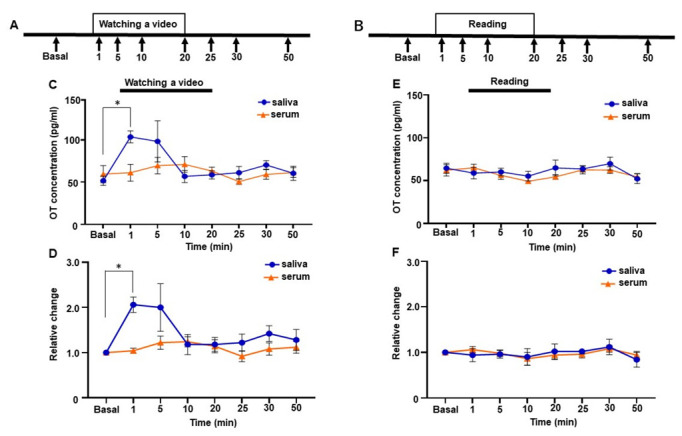
Changes in salivary and serum oxytocin (OT) concentrations of one woman while watching the video of the infant or reading a newspaper. (A and B) The time course of the video (**A**) and newspaper-reading experiments (**B**). (**C**) Salivary and serum OT concentrations while watching the video. (**D**) Relative changes in salivary and serum OT concentrations while watching the video. (**E**) Salivary and serum OT concentrations while reading a newspaper. (**F**) Relative changes in salivary and serum OT concentrations while reading a newspaper. The experiment was repeated six times with one mother (n = 6). Values were averaged across six repeated experiments, respectively. Values are the mean ± SEM; one-way repeated measures ANOVA (d.f. = 7; * *p* < 0.05).

**Figure 6:**
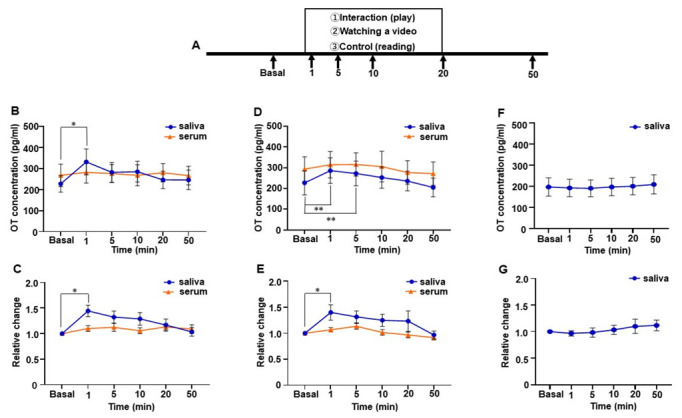
Changes in salivary and serum oxytocin (OT) concentrations of a group of mothers. (**A**) Schematic procedure of the direct mother-infant social interaction, video-watching, and reading tests. (**B**) Salivary and serum OT level during the direct mother-infant social interaction test. (**C**) Relative change in salivary and serum OT during the direct mother-infant interaction test. (**D**) Salivary and serum OT level while watching the video of the infants. (**E**) Relative change in salivary and serum OT while watching the video of the infants. (**F**) Salivary OT level while reading a newspaper or a book. (**G**) Relative change in salivary OT while reading a newspaper or a book. Nine participants took part in the direct mother-infant interaction test, and six of these took part in the video and newspaper or book reading test. Values are given as the means ± SEM; one-way repeated measures ANOVA (d.f. = 9, interaction test; d.f. = 7, video and reading test; * *p* < 0.05, ** *p* < 0.01).

All samples were collected either at 13:00–15:00 during pregnancy (Figure 1A) or 9:00–11:00 a.m. postpartum (Figure 2, Figure 3, Figure 4, Figure 5 and Figure 6). The participant was instructed to refrain from consuming food and drink (other than water), from numerous activities, and stress for at least 1.5 h before the basal sample collection. During parturition, samples were collected from the beginning of delivery to the end of the first trimester of labor intervals and at seizures (Figure 1B). Saliva samples were collected before blood sampling each time. For saliva samples, about 2 mL of spontaneous drool was collected in a 15-mL conical tube. Samples were kept on ice until the end of each experiment and stored at −80 °C until assayed. At the time of assay, samples were thawed naturally and then centrifuged at 4 °C, 15,000× *g* for 15 min. Supernatants were collected and used for the assay. For serum and plasma sampling, 9 mL of blood were drawn each time to the vacutainer either in a serum- or a plasma-separator tube (Venogect II, TERUMO, Tokyo, Japan). For sampling blood at multiple time points, an indwelling needle was placed in a forearm vein. Collected blood samples were stored in a refrigerator at 4 °C for 10 min and then centrifuged at 4 °C, 1500× *g* for 20 min after collecting all samples from each time point. Supernatants were collected and frozen at −80 °C until assayed. All samples were thawed only once and assayed.

### 2.3. Procedures for Sampling during Breastfeeding

Samplings during breastfeeding were performed on postpartum days 1, 3, 5, 7, 30, and 90. At least a 2 h gap after the previous feeding occurred prior to breastfeeding sampling, and the participant and infant had not interacted for at least 30 min. The following time points were taken at the time of breastfeeding, 5 min before, 5 and 10 min after initiation of breastfeeding, and 30 min after breastfeeding ended (Figure 2A). While waiting during the 30-min interval, the participant read newspapers. The time points were determined according to the half-life of plasma OT [42] and previous studies that showed the elevated OT level in the plasma during breastfeeding [43,44].

### 2.4. Social Interaction Test

At the participant’s home, the test was repeated seven times at 6 months postpartum (Figure 4A). Participant and infant interaction was avoided for at least 30 min prior to the interaction test. Then, for 20 min, the participant and infant interacted via their normal touch and play. Immediately after the interaction, the participant separated herself from her infant and waited for 30 min before beginning to breastfeed. While waiting, the participant read either newspapers or books. Samples for basal time points were taken 5 min before the interaction and then collected at the following time points during the interaction: 1, 5, 10, and 20 min. After 30 min of waiting, the samples were collected, and the participant then immediately started breastfeeding. Next, samples were collected 5 and 10 min after breastfeeding began and 30 min after it ended. One serum dataset was excluded because of a missing value caused by a technical problem. The interaction test was also conducted in the group of mothers during the 6 months postpartum period using the same procedure and time course, except that the breastfeeding test did not follow (Figure 6A).

### 2.5. Video Test

The video test was repeated six times at 11 months postpartum and performed in the participant’s home (Figure 5A). Prior to the video tests, we asked the participant to video her infant. We edited these videos and mounted a 20 min video clip. At each of the six tests, the video clip was new to the participant. Since the participant has three other children, the video did not always contain only images of the infant. On video test days, the participant did not have contact with her infant for at least 30 min before the video test. Samples for the basal time point were taken 5 min before the video test. Then, samples were collected at the following time points after starting the video: 1 min, 5 min, 10 min, 20 min, 25 min, 30 min, and 50 min. While waiting 30 min after watching the video, the participant read newspapers. As a control experiment that was repeated six times, the participant read newspapers for 20 min instead of watching videos (Figure 5B). The same experimental procedure was conducted with six mothers (6–7 months postpartum) who participated in the social interaction test for the video and the control test (Figure 6A). The samples at 25 and 30 min time points were not taken, as no significant changes were observed in OT concentrations at these time points in the subject of the trajectory study.

### 2.6. OT Extraction

Solid-phase extraction was performed according to the manufacturer’s protocol using 500 mg Sep-Pak C18 cartridges (Waters Corporation, Milford, MA, USA) for serum samples (Supplementary Figure S1). Briefly, columns were equilibrated with 2 mL of acetonitrile, then twice with 7 mL of 0.1% trifluoroacetic acid (TFA). Up to 500 μL of plasma was mixed with an equal volume of 0.1% TFA and centrifuged at 15,000× *g* for 15 min at 4 °C; the acidified and clarified plasma was then applied to the column. The flow-through fraction was discarded and columns were washed three times with 7 mL of 0.1% TFA. OT was eluted with 3 mL of 95% acetonitrile. The solvent was then evaporated using a Refrigerated CentriVap Vacuum concentrator (LABCONCO, Kansas City, MO, USA) and completely dried by lyophilization.

### 2.7. OT Quantification

Serum and salivary OT values were measured using a 96–plate commercial OT-ELISA kit (CAT. NO; ADI-901-153A-0001, Enzo Life Sciences, Farmingdale, NY, USA), as previously reported [29,30]. Detection sensitivity was 15 pg/mL, and the measurement range was 15.6–1000 pg/mL. Measurements were performed in duplicate. The optical density of the samples and standards was measured at wavelengths of 405 and 590 nm by a microplate reader (Bio-Rad, Richmond, CA, USA). Sample OT concentrations were calculated by relevant standard curves, as instructed in the manufacturer’s protocol. Observed intra-assay and inter-assay coefficients of variability were <2.4% and <3.8%, respectively.

### 2.8. Statistical Analysis

Statistical analysis was performed using Prism 8 software (GraphPad Software Inc., San Diego, CA, USA). Data are represented as the mean ± SEM. Repeated measures ANOVA was conducted to examine whether the pattern of change in OT differed by the time of feeding and interaction with the infant. Correlation coefficients were used to determine any correlation between serum and salivary OT. In all analyses, *p* < 0.05 indicated statistical significance.

## 3. Results

We conducted a trajectory study following one woman’s peripheral OT level. OT concentrations during pregnancy, parturition, and breastfeeding were monitored in both serum and saliva.

### 3.1. OT Levels of One Woman during Pregnancy

Salivary OT concentration gradually increased toward 40 weeks of pregnancy, while serum OT concentration showed a sharp increase at 26 weeks and slowly declined toward 40 weeks (Figure 1A). In saliva, the peak was observed in the third trimester (40 weeks), and in the second trimester (26 weeks) in the serum (Figure 1A). Individual concentration varied from 38.2 to 109.7 pg/mL for saliva and 76.3 to 275.2 pg/mL for serum (Figure 1A). Salivary and serum OT levels increased by approximately threefold (saliva, 2.9-fold; serum, 3.6-fold) compared to those at 6 weeks of pregnancy (Figure 3B).

### 3.2. OT Levels during Parturition of One Woman

In both saliva and serum, OT levels were slightly higher during uterine contractions than during intermittent periods of labor in all terms (Figure 1B). While the saliva concentration showed a gradual increase toward the second stage of parturition (ranging from 63.3 to 208.1 pg/mL), the serum OT level exhibited only small changes (from 132.0 to 195.5 pg/mL). The fold increase in salivary concentration from the cervix 3 cm phase to the cervix 8 cm phase was 2.1 for the latent phase and 2.2 for the contraction phase. Serum OT increased 1.2-fold for the latent phase and 1.3-fold for the contraction phase. These results indicate that changes in OT concentration were more obvious in saliva.

### 3.3. Changes in Salivary and Serum OT Levels during Breastfeeding of One Woman

Salivary and serum OT levels during breastfeeding were monitored on postpartum days 1, 3, 5, 7, 30, and 90. Samples were taken before breastfeeding (basal), at 5 and 10 min after initiation of breastfeeding, and 30 min after the end of breastfeeding (Figure 2A). Significant increases in both salivary and serum OT concentrations during breastfeeding were detected by one-way repeated measures ANOVA (saliva, *F* [1.395, 6.973] = 11.83, *p* = 0.008; serum, *F* [2.029, 10.15] = 4.547, *p* = 0.039). Tukey’s multiple comparison test revealed a significant increase in saliva OT concentration from 5 min, sustained up to 10 min (basal 38.2 ± 6.3 pg/mL vs. 5 min 71.4 ± 5.9 pg/mL, *p* < 0.01; basal vs. 10 min 73.3 ± 9.3 pg/mL, *p* = 0.040). Then, OT levels decreased to the basal level 30 min after breastfeeding ended, differing significantly from the 10 min point (10 min vs. 30 min 54.6 ± 8.8 pg/mL, Tukey’s multiple comparison test, *p* = 0.018) (Figure 2B).

In contrast, serum OT levels increased slightly at 5 min (basal 69.6 ± 12.2 pg/mL, 5 min, 82.1 ± 8.3 pg/mL), increased to 104.7 ± 17.9 pg/mL at 10 min, and decreased to 77.7 ± 13.4 pg/mL 30 min after breastfeeding ended. Tukey’s multiple comparison test showed a significant increase in serum OT level at 10 min (basal vs. 10 min, *p* = 0.049) (Figure 2C). The fold increase at 10 min in saliva and serum were 2.1 ± 0.4 and 1.5 ± 0.1, respectively. In other words, the increase in OT saliva concentration was more obvious from an earlier time point during breastfeeding than in OT serum concentration.

### 3.4. Trajectory of Salivary and Serum OT Levels during Pre- and Postpartum Periods of One Woman

Figure 3A illustrates the trajectory of salivary and serum OT concentrations from early pregnancy to postpartum day 90. To compare changes in OT concentration from pre- to post-delivery of one woman, OT values in Figure 1 were used. For the parturition period, the values were used during intermittent labor at 3 cm of cervix opening. For the postpartum period, we plotted basal values from each monitored postpartum day. Figure 3B replots to show the relative changes in salivary and serum OT levels at various time points during pre- and postpartum periods.

The peak salivary OT concentration was at 40 weeks (Figure 3A), 2.9-fold higher than during early pregnancy (6 weeks, Figure 3B). Conversely, the peak serum OT level was detected during the second trimester at 26 weeks (Figure 3A), 3.6-fold higher than during early pregnancy at 6 weeks (Figure 3B).

Interestingly, both salivary and serum OT levels declined at the beginning of labor (Figure 3A,B); although, concentrations increased up to 2.2-fold for salivary OT and 1.3-fold for serum OT during the contraction period of the cervix at 8 cm, compared to the contraction period of the cervix at 3 cm (Figure 1B). Then, salivary and serum OT levels appeared to decline quickly after labor. While salivary OT concentration returned to the early pregnancy level on postpartum day 1, the serum OT level dropped below the early pregnancy level on day 3. The serum OT concentration stayed lower than the early pregnancy level for the first postpartum week and returned to the early pregnancy level by postnatal day 30 (Figure 3A,B). Concentrations of salivary and serum OT from early pregnancy to postpartum day 90 showed a significant positive correlation (Pearson’s correlation coefficient *r* = 0.509, *p* < 0.001, Figure 3C).

### 3.5. Social Interaction Test by One Subject of Trajectory Study

Parent-infant contact changes OT levels both in parents and infants; mothers who show more affectionate contact also show higher increases in salivary oxytocin [12]. However, how OT levels change during contact was not investigated previously. Therefore, we collected saliva and serum samples from early time points, i.e., 1, 5, 10, and 20 min, during mother-infant direct social interaction to monitor changes in OT level. Furthermore, to assess whether changes in OT concentration were due to social interaction, we monitored the OT level during breastfeeding (Figure 4A). The sampling for breastfeeding started 30 min after the social interaction, as the half-life of OT in the blood is approximately 30 min [42]. Surprisingly, OT in saliva showed a sharp increase during social interaction, while OT in serum did not (Figure 4B,D). One-way repeated measures ANOVA showed a significant difference in salivary OT levels (*F* [2.436, 12.18] = 6.830, *p* = 0.008, Figure 4B). Post hoc analysis using Tukey’s multiple comparison test indicated significant increases in the OT level at 1 and 5 min compared with the basal level (basal 49.1 ± 5.7 pg/mL vs. 1 min, 97.2 ± 4.6 pg/mL, *p* = 0.043; vs. 5 min, 91.6 ± 7.9 pg/mL, *p* = 0.038). Peak OT concentration could be detected at 1 min, which was 2.1 ± 0.2-fold higher than at the basal level; thereafter, it decreased gradually (Figure 4C).

When we monitored salivary OT concentration during breastfeeding after a social interaction period, the increase was observed at 5 min after initiation of breastfeeding (55 min time points, Tukey’s multiple comparison test, *p* = 0.314), and the OT level remained high for 10 min. However, this increase was not significant compared to the initiation of breastfeeding (50 min time points, Tukey’s multiple comparison test, *p* = 0.570).

In contrast, serum OT levels did not reach significance but remained at the same level during social interaction (*F* [2.556, 10.22] = 3.403, *p* = 0.066], Figure 4D). Breastfeeding conducted 30 min after the interaction’s end showed a significant increase in serum OT levels when compared before (50 min) and 5 min after (55 min) initiation of breastfeeding (Tukey’s multiple comparison test [50 min 69.8 ± 9.1 pg/mL vs. 55 min 107.4 ± 9.3 pg/mL, *p =* 0.015]). 

We analyzed the relative changes in saliva and serum OT. Saliva OT exhibited a significant difference over time when analyzed by one-way repeated measures ANOVA (*F* [2.455, 12.27] = 7.908, *p* = 0.005, Figure 4C), whereas serum OT did not (*F* [1.865, 7.458] = 2.509, *p* = 0.148, Figure 4E). Though the relative changes in the 1 and 5 min time points showed a nearly 2.0-fold increase compared to the basal level in saliva, the post hoc analysis using Tukey’s multiple comparison tests did not reach statistical significance (basal vs. 1 min, *p* = 0.101, vs. 5 min, *p* = 0.057).

### 3.6. Video Test by One Subject of Trajectory Study

The mother’s salivary OT level increased during physical-contact social interaction with her infant. This effect might be mediated by visual, auditory, and/or tactile sensations. Therefore, we measured salivary and serum OT levels by showing a video of her infant for 20 min to see if visual or auditory stimuli alter OT levels. Because the changes in OT values while mothers are recalling their infants have not been examined elsewhere, we increased the sampling points to capture the changes in OT concentration. The time points for evaluation were 0, 1, 5, 10, 20, 25, 30, and 50 min (Figure 5A). Immediately after 1 min had elapsed from the beginning of the video, the mother’s salivary OT levels increased and were highly maintained for 5 min (basal 51.7 ± 5.5 pg/mL, 1 min 103.5 ± 7.0 pg/mL, 5 min 98.2 ± 24.3 pg/mL, Figure 5C). Then, salivary OT levels decreased to the basal level after 10 min (10 min 56.8 ± 7.2 pg/mL). Although one-way repeated measures ANOVA did not reach significance in salivary OT levels (*F* [1.825, 7.300] = 4.049, *p* = 0.068]), post hoc analysis with Tukey’s multiple comparison test showed significant difference at baseline and 1 min after the initiation of video-watching (basal vs. 1 min, *p* = 0.010). In contrast, the mother’s serum OT levels showed no great change while watching the video. One-way repeated measures ANOVA revealed no significant difference in serum OT levels (*F* [2.488, 9.950] = 1.875, *p* = 0.201]). 

We then analyzed the relative changes in OT levels in saliva and serum. The changes did not reach significance over time when analyzed by one-way repeated measures ANOVA (*F* [1.744, 6.977] = 4.073, *p* = 0.071, Figure 5D), although post hoc analysis with Tukey’s multiple comparison test showed a significant difference between basal values and those taken 1 min after the initiation of video-watching (basal vs. 1 min, *p* = 0.028). 

In a control test, neither the mother’s saliva OT nor her blood OT changed significantly while she was reading the newspaper (saliva, *F* [2.448, 9.791] = 0.938, *p* = 0.442]) (serum, *F* [2.674, 10.70] = 2.246, *p* = 0.145]), (Figure 5E). No significant changes were observed in the relative changes in serum (*F* [2.538, 10.15] = 1.789, *p* = 0.214, Figure 5D) or in both saliva and serum in the control conditions (saliva, *F* [2.460, 9.838] = 0.852, *p* = 0.477, serum, *F* [2.342, 9.368] = 1.536, *p* = 0.267, Figure 5F).

### 3.7. Social Interaction Test by a Group of Mothers

Because we found that only saliva and not serum OT increased in the social interaction test, we conducted the same experiment with a group of mothers. We assessed nine participants and detected a rapid increase in OT in saliva during the mother-infant social interaction test, but no increase was observed in serum OT (Figure 6B). A one-way repeated measures ANOVA followed by post hoc analysis using Tukey’s multiple comparison test revealed a significant difference in salivary OT levels (*F* [3.197, 25.57] = 4.082, *p* = 0.016) and between basal values and those measured at 1 min (basal; 227.7 ± 39.3 pg/mL vs. 1 min; 332.0 ± 61.7 pg/mL, *p* = 0.045). As with the result for the single subject, the peak was at 1 min (1.4 ± 0.1-fold higher than the basal level) and, thereafter, decreased gradually (Figure 6C). The relative change in salivary OT also showed a significant difference in salivary OT (one-way repeated measures ANOVA (*F* [3.338, 26.70] = 4.046, *p* = 0.014) and post hoc analysis using Tukey’s multiple comparison test revealed the significance between the basal and 1 min time points (*p* = 0.033) (Figure 6C). No difference was detected in the relative changes in serum OT during the social interaction test.

### 3.8. Video Test by a Group of Mothers

We conducted the video and control (reading) tests with a group of mothers. Six of the nine mothers who participated in the social interaction test participated in the video and control (reading) tests. As with the result of the single subject’s video test, a group of mothers showed a rapid increase in saliva OT but not in the serum. A one-way repeated measures ANOVA showed significant differences in salivary OT concentrations (*F* [1.846, 9.231] = 9.921, *p* < 0.01). The post hoc analysis using Tukey’s multiple comparison test showed significant differences between basal, 1 min, and 5 min time points (basal: 226.7 ± 57.9 pg/mL vs. 1 min: 285.8 ± 61.4 pg/mL, *p* < 0.01, vs. 5 min: 272.3 ± 59.5 pg/mL, *p* < 0.01, Figure 6D). The relative change of salivary OT also revealed a significant difference (*F* [1.708, 8.539] = 5.277, *p* = 0.036) and the post hoc analysis using Tukey’s multiple comparison test showed a significant increase at the 1 min time point (basal vs. 1 min, *p* = 0.026, 1.4 ± 0.1-fold higher than the basal level, Figure 6E). Again, no difference was detected in relative changes in the serum OT during the video test. Additionally, the OT level in saliva remained almost the same over the time during the control test, in which they do not recall their children (*F* [1.986, 9.932] = 0.421, *p* = 0.667). Taken together, mothers within 1 year of post-pregnancy showed a rapid rise in saliva OT but not in serum OT while interacting with their infants or in a situation that reminded them of their infants.

## 4. Discussion

### 4.1. OT Concentration during the Pregnancy and Labor of One Mother

We followed the trajectory of OT levels in the serum and saliva during pregnancy and labor of one subject. Studies examining the plasma OT level during birth were reported from the 1970s to the 1990s [41], but now, replicating a similar study is difficult for practical and ethical reasons. In that sense, our study is noteworthy for following changes in both salivary and serum OT levels.

In this study, both salivary and serum OT levels increased during pregnancy and labor. However, while salivary OT concentrations showed a sustained increase during pregnancy and labor, serum OT did not show identical changes. One reason for the differing trajectories might be due to the different half-life of OT. Thus, further studies are necessary to examine degrading enzymes’ activity of and/or OT-binding molecules in both serum and saliva.

During pregnancy, the peak serum increase was observed at mid-pregnancy. Similar to previous measurements in plasma (2.5- to 4.0-fold) [45,46], the increase from early pregnancy to the peak was about 3.6-fold in serum. In general, OT concentrations in blood samples increase as gestational age proceeds [41], but trajectory patterns may vary among individual subjects, as Levine et al. reported [47]. Therefore, we cannot say that the OT concentration’s trajectory during our subject’s pregnancy is the typical case or specific to her. Furthermore, during the parturient period, serum OT did not change markedly after the active phase (uterine cervix 5 cm). In a previous report, the level of plasma OT during the first term is rather stable, and a significant increase was observed during the birth of the fetal head [48]. Our subject’s serum OT concentration might have increased significantly after the later time points of labor, but we did not measure them in this study.

Highly possibly, the increase in serum OT concentration during pregnancy and labor may be affected by the level of aminopeptidases, such as placental leucine aminopeptidase (P-LAP), which degrades OT to maintain blood OT concentration during the perinatal period [49]. Reportedly, the P-LAP serum level increases with gestation and reaches its maximum near the end of pregnancy [50]. Decreased P-LAP activity is observed in sera of patients with spontaneous preterm delivery [50]. Therefore, P-LAP may prevent the premature onset of uterine contraction by degrading OT and play a role in the maintenance of normal pregnancy [39]. In contrast, individual differences in OT levels’ trajectory during this period may not only be due to OT-degrading enzymes’ activity but to the Receptor for Advanced Glycation Products (RAGE) as well [51,52,53].

### 4.2. OT Concentration during Breastfeeding of One Mother

In our serum samples of one mother, the increase rate was similar to those in the previous report [54,55]. However, to the best of our knowledge, there is no study examining the changes of OT concentration in a short time window during breastfeeding in saliva samples. The peak increase compared to the basal was 1.5-fold for serum and 2.1-for saliva. In both samples, increases were detected during 10 min of breastfeeding, but saliva samples showed a clearer change. Such a difference in OT level may simply reflect the different half-life of OT. Additionally, given the direct interaction test results, the salivary concentration might have increased not only due to the stimulus of breastfeeding but also to the stimuli of direct interaction.

### 4.3. Salivary, Not Serum OT, Increased during Mother-Infant Social Interaction in One Mother of Trajectory Study and the Group of Mothers

In the present study, we aimed to detect any differences in the mother’s OT level when she is with her infant or when she is just recalling her infant from the video she has been watching. The participants in the trajectory study showed increased OT in saliva but not in the serum in both conditions. This result was replicated in the group of mothers as well. In both test conditions, a rapid increase in salivary OT concentration was detected and all of the participants showed a peak either at the 1 or 10 min time point. The subject in the trajectory study showed a peak in OT concentration at 1 min in three of six times during the social interaction test and three of five times during the video test. Of the group of mothers, 55.6% and 50.0% showed a 1 min peak in the social interaction test and the video test, respectively. Further, the OT concentration dropped to the basal level by the 20 min time point in all mothers. Taken together, our findings suggest that the maternal OT response occurs rapidly when it is associated with infants. Additionally, one should pay attention to the time course of sampling when examining the OT response in mothers in a context related to their infants.

To check whether the increased OT concentration was not detectable because we used the serum, we conducted the same video test to collect plasma samples in the subject of the trajectory study. Although salivary OT showed a significant increase (Student *t*-Test, n = 4 sets, *p* < 0.05), plasma OT did not show any significant increase at any time points (Supplementary Figure S1). Therefore, an increased blood OT concentration was not detected regardless of the use of plasma or serum samples. This result follows that of Grewen et al., who also did not detect a significant increase in maternal plasma OT during 5 min of direct contact with the infant [55].

### 4.4. Saliva OT as Surrogate for Central Release of OT

It is of general interest whether peripheral OT concentration can be used as an indicator of central OT level. However, due to the difficulty of collecting CSF, comparison studies on peripheral and CSF OT levels are unlikely to proceed in humans. Therefore, it is important to conduct a study to associate changes in peripheral OT levels with human behavior. In the present study, increased maternal salivary OT was not only observed during mother-infant interactions but also when mothers were recalling their infants. A recent study has shown a correlation between CSF and salivary OT concentrations [38]. Therefore, changes in saliva OT concentration may reflect the central release of OT, and this may be a valid surrogate value for CSF OT concentrations. Further substantial studies are necessary to assess whether and when salivary OT concentrations are associated with human behavior or emotion.

## 5. Limitations of the Study

We followed the OT levels in serum and saliva during the pregnancy, labor, and lactation period in one woman. Our preliminary study is the first attempt to simultaneously collect blood and saliva samples from term to term in the same individual. We believe that examining how OT fluctuates throughout the term in each individual may give us more insight into the use of OT as a treatment for psychiatric disorders, including postpartum depression. However, due to the recent ethical restrictions and the current global situation of the COVID-19 pandemic, we only obtained the data from a single subject. Further studies in large populations need to be performed to generalize the data obtained from a single subject.

The exact mechanism of how OT is transported in the saliva is not well understood. Saliva is a product of the combination of salivary glands in the oral cavity and components derived from the blood by passive diffusion or active transport [56]. Diffusion through a concentration gradient is the classic route for lipid-soluble steroids, amines, or hydrophilic peptides, although no studies have confirmed whether this is so for OT. Our simultaneous sampling study of blood and saliva revealed a context-dependent difference in the pattern of OT fluctuation. Further studies are required to understand how OT is transported in saliva and the turnover mechanism of saliva OT.

In the current study, ELISA was used to measure saliva and serum OT concentrations. One issue of using ELISA is the specificity of the antibody. Antibodies detect not only the target analyte’s free form, but they may also detect different forms, such as precursors or metabolites, or forms within other protein complexes [51,57]. These phenomena may partly explain the differences in the OT concentration detected by ELISA kits and/or by HPLC-MS or radioimmunoassay. In fact, the range we detected was one order of magnitude higher than that reported in other studies [26,27]. However, the fold change in OT concentrations was similar to previous studies with perinatal women. The fold changes in plasma OT concentration during pregnancy in previous studies (2.5–4.0 fold) [45,46] were also similar to that in our study (2.9-fold). Therefore, the monitored values may not reflect the absolute physiological values of free OT, but relative changes can be compared.

The subjects of our study are solely Japanese. It is reported that parents’ attitudes toward parenting and nursing may differ depending on their background, for example, culture, religion, customs, race, personality, mental illness, and social status. Further study is necessary to assess whether psychosocial factors affect the fluctuation of OT response.

## 6. Conclusions

The assessment of salivary and serum changes in OT concentration across pregnancy to postpartum in one subject revealed that the two change similarly during pregnancy, labor, and breastfeeding. By contrast, only the salivary OT level changed rapidly during direct mother-infant interaction and the video test. These results were replicated in a small number of mothers. Although further study with a larger subject group is necessary, our results indicate that the salivary samples may reflect not only the mother’s physical but also her emotional state. Thus, we propose that saliva may be more sensitive for monitoring a woman’s OT levels during pre-and postpartum periods. Additionally, examining the relative changes in OT levels in an experimental or stimulated context may be a more efficient way of distinguishing differences in endogenous OT activity rather than checking the basal level of OT. Detecting dysregulated OT response in the stimulated condition may be useful for the diagnosis or treatment of patients with psychiatric disorders, especially maternal depression.

## Figures and Tables

**Table 1:** Demographic and obstetric characteristics.

		n (%)	Mean ± SD	Min.	Max.	n (%)	Mean ± SD	Min.	Max.
		Social interaction test (n = 9)				Video test (n = 6)			
Mother Age (years)			32.3 (±4.8)	26	39		32.0 (±5.3)	26	39
Infant Age	6 months	9 (100.0)				4 (66.7)			
	7 months	-				2 (33.3)			
Obstetric History	Primipara	4 (44.4)				4 (66.7)			
	Multipara	5 (55.6)				2 (33.3)			
Reproductive History	Naturally	6 (66.7)				4 (66.7)			
	Infertility treatment	3 (33.3)				2 (33.3)			
Education	<4 years college graduate	-				-			
	Graduated 4 years college	6 (66.7)				4 (66.7)			
	Postgraduate	3 (33.3)				2 (33.3)			
Family Type	Nuclear family	7 (77.8)				6 (100.0)			
	Extended family	2 (22.2)				-			
Employment Situation	Working	-				-			
	Maternity leave	7 (77.8)				4 (66.7)			
	Housewife	2 (22.2)				2 (33.3)			
Infant Birth Weight			3052 (±322.4)	2680	3620		3139 (±306.5)	2728	3620
Menstruation	Postpartum Amenorrhea	7 (77.8)				4 (66.7)			
	Restart	2 (22.2)				2 (33.3)			

## Data Availability

The data presented in this study are available on request from the corresponding author. The data are not publicly available due to ethical reason.

## Supplementary Materials

The following supporting information can be downloaded at: https://www.mdpi.com/article/10.3390/brainsci12091246/s1, Figure S1: Changes in saliva, serum, and plasma oxytocin (OT) concentrations of one woman while watching the video of the infant.
