# Supplementary material for: Infant Stimulation Induced a Rapid Increase in Maternal Salivary Oxytocin
Source: Brain Sci. 2022 Sep 15;12(9):1246. doi: 10.3390/brainsci12091246 (PMC9497188; doi:10.3390/brainsci12091246)
Supplement: Supplementary file 1 [file brainsci-12-01246-s001.zip › brainsci-1838757-supplementary.pdf]

Suppl. Fig. 1

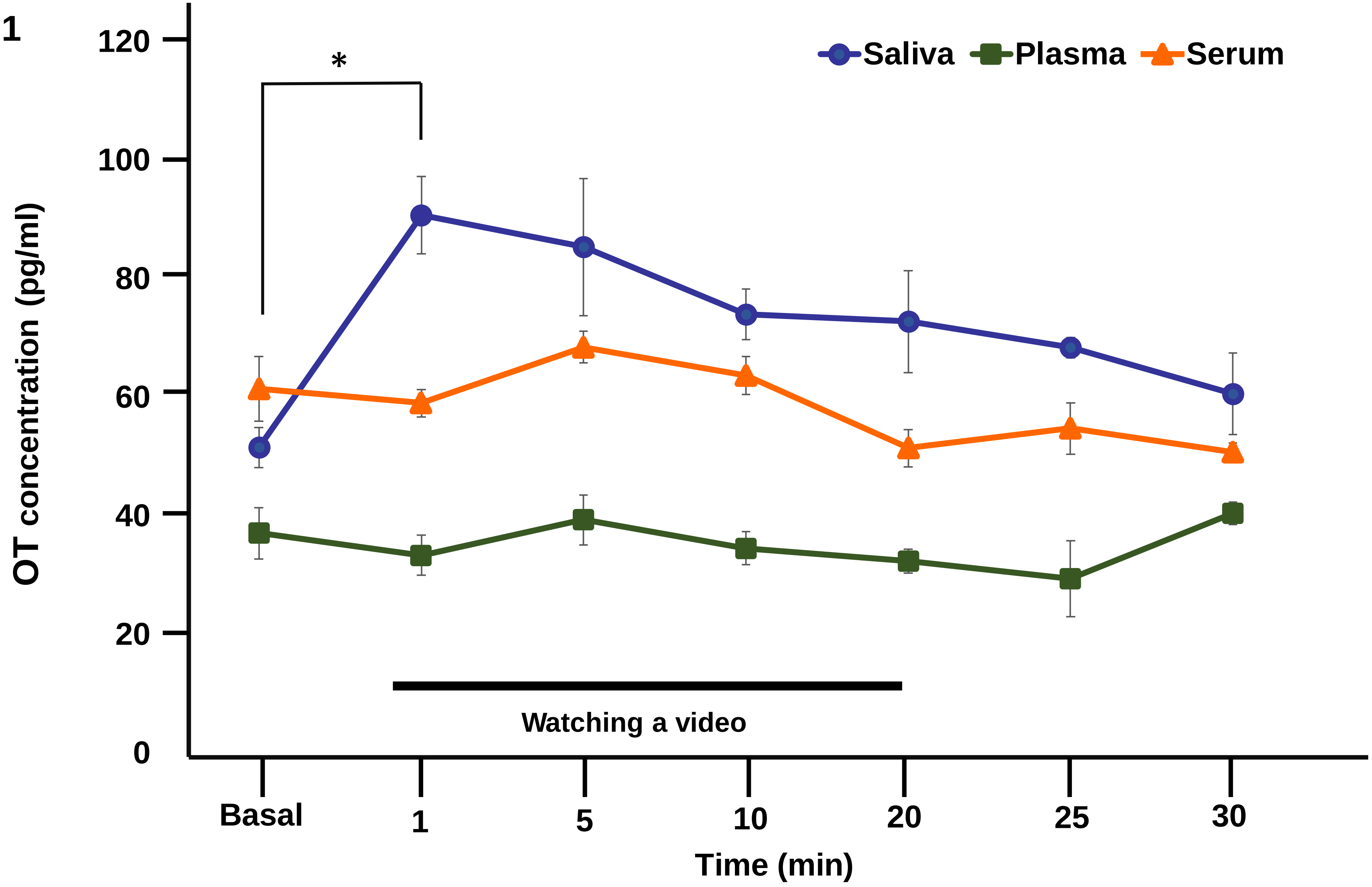

Changes in saliva, serum, and plasma oxytocin (OT) concentrations of one woman while watching the video of the infant.
